# Painful vertical diplopia as a presentation of a pituitary mass

**DOI:** 10.1186/1471-2415-7-4

**Published:** 2007-03-15

**Authors:** Shveta Bansal, Kaveri Mandal, Ahmed Kamal

**Affiliations:** 1 Department of Ophthalmology, Royal Liverpool University Hospital, UK; 2 Department of Ophthalmology, Walton Hospital, Liverpool, UK

## Abstract

**Background:**

Pituitary tumours may present with a variety of neurological and endocrinological signs and symptoms. It is very rare however for them to present with sudden onset painful diplopia. The current literature and possible mechanisms for this are discussed.

**Case presentation:**

We describe a case of a pituitary mass which presented with sudden onset painful diplopia with an associated restricted pattern on Lees Chart testing. This led to an initial working diagnosis of orbital myositis.

**Conclusion:**

Awareness of different modes of presentation of pituitary lesions is important so that appropriate imaging may be requested and delay in diagnosis prevented.

## Background

It is rare for a pituitary tumour to present with painful diplopia due to a partial third nerve palsy. We present such a case in which the initial lack of pupil involvement or ptosis combined with the restricted painful ocular movements, led to an initial suspected diagnosis of orbital myositis. This is a previously undocumented mode of presentation of a large non-secretory pituitary mass. The history is unusual in its acute, painful onset and examination findings were in keeping with a restricted ocular motility defect.

## Case presentation

**Figure 1:**
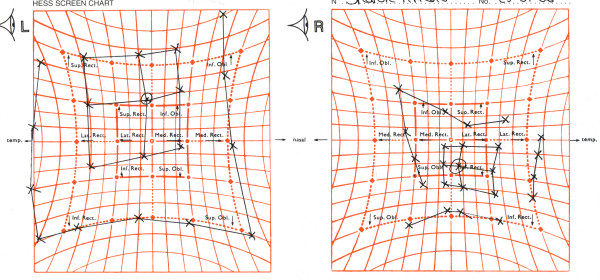
Lees Chart showing a restrictive profile.

A 47 year old female who was otherwise healthy presented with a sudden onset vertical diplopia associated with right sided headache and right eye pain. On examination she had a right hypotropia measuring 20^ for distance. Eye movements were limited in dextroelevation with associated discomfort. Ocular motility testing with a Lees chart showed a restrictive profile (figure 1). There was no proptosis, ptosis or lid retraction. Snellen visual acuities were 6/5 in each eye. Colour vision and visual fields were normal. Anterior segment and fundal examinations were unremarkable.

**Figure 2:**
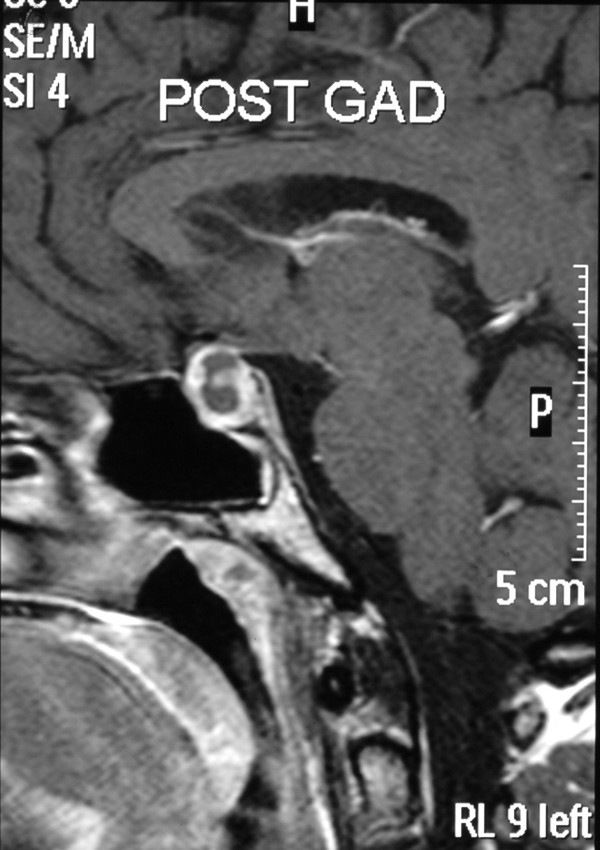
Magnetic resonance T1-weighted contrast-enhanced imaging; sagittal view.

**Figure 3:**
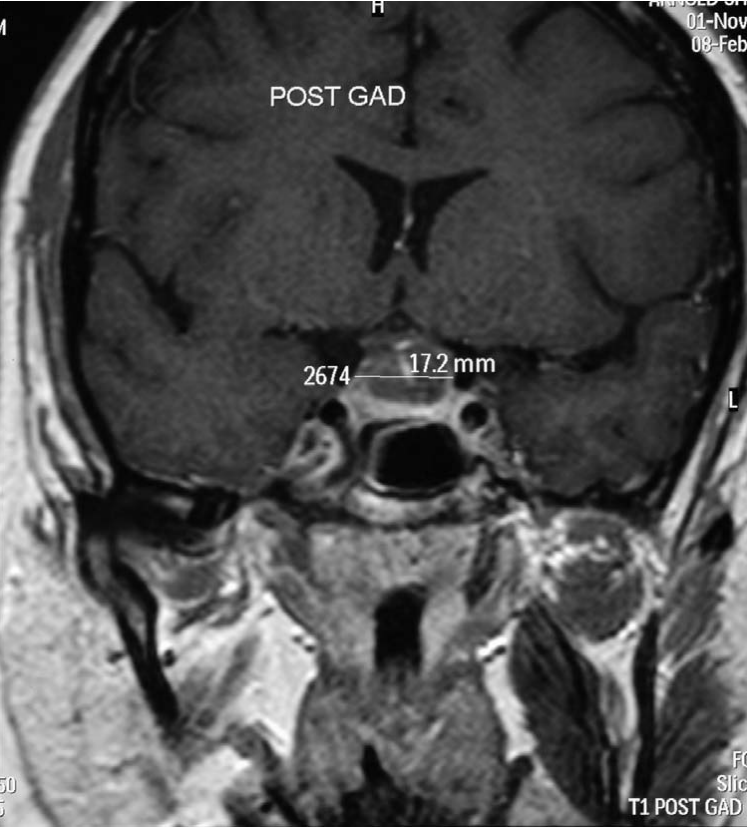
Magnetic resonance T1-weighted contrast-enhanced imaging; coronal view.

Thyroid function tests and inflammatory markers were within normal limits (Free T4 14.2 pmol/L, Free T3 5.9 pmol/L, TSH 1.48 mu/L, C-Reactive Protein 13 mg/L). An initial diagnosis of orbital myositis was suspected due to the marked painful limitation in elevation of the right eye. MRI of the orbits revealed no muscle or tendon abnormality but an incidental pituitary lesion was identified. Further intracranial imaging with contrast delineated a cystic pituitary mass compressing the chiasm which showed ring enhancement of the tumour, measuring 1.72 cm × 1.28 cm × 1.29 cm (figures 2, 3). There was no radiological evidence of any intracerebral aneurysm or of invasion into the cavernous sinus.

Pituitary hormone levels and serum osmolality were within normal limits (Prolactin 446 miu/L, Testosterone 1.7 nmol/L, FSH 13.0 iu/L, LH 6.4 iu/L, Growth Hormone 1.19 miu/L, IGF 1 22 nmol/L, Serum Osmolality 297 mosm/kg).

The following week the patient developed anisocoria, slight right sided ptosis and her visual acuities fell to 6/12 right eye and 6/9 in the left eye. Although initially a macroadenoma or craniopharyngioma was suspected, further histological analysis revealed a benign squamous epithelium lined lesion which was consistent with an epidermoid cyst, dermoid cyst or a teratoma. There were no signs of infarction or haemorrhage. The lesion was removed by transphenoidal resection, leading to a gradual resolution of symptoms.

There have been reports of recurrent or intermittent third nerve palsy as well as ptosis as a presenting feature of pituitary tumours [1-4]. However, an acute presentation with pain on eye movements and a restricted ocular motility pattern has not been previously documented. A large pituitary mass may compress any of the cranial nerves within the lateral wall of the cavernous sinus however this tends to be late in the course of tumour growth [3]. The third and fourth cranial nerves are more susceptible as the abducent nerve affords some protection from the internal carotid artery. Direct invasion of the tumour through the sinus wall may also occur. Mechanical compression of the oculomotor nerve against the unyielding interclinoid ligament of the cavernous sinus wall tends to bring about a slow onset paralysis. Rapid onset of third nerve paralysis has been attributed to compromise of the vascular supply to the nerve [4], due to compression of the vasa nervorum originating in the internal carotid artery [5]. Sudden symptoms have also been seen in pituitary apoplexy [6], where immediate treatment with bromocriptine and steroids has been advocated, on the basis that most macroadenomas are prolactinomas [7]. Finally, the possibility of a coincidental pituitary tumour and a spontaneously recovering micro-infarctive third nerve palsy must also be considered.

## Conclusion

In our case there was an initial sudden onset of vertical diplopia which may have been the result of compromise to the vascular supply of the third nerve. Pupil involvement eventually followed. On radiological analysis the tumour appeared to abut the optic chiasm however visual fields remained full. Although this presentation is unusual, a pituitary mass should be included in the differential diagnosis in cases of isolated painful diplopia.

## Competing interests

The author(s) declare that they have no competing interests.

## Authors' contributions

SB – The lead author involved in carrying out the literature search, study design and writing the case report.

KM assisted with writing the paper, supervising and designing the study.

AK supervised the management of the case and participated in its design and writing the case report.

All authors have been involved in approving the final manuscript.

## Pre-publication history

The pre-publication history for this paper can be accessed here:


